# Clinical and Molecular Heterogeneity of *MYH3*-Related Arthrogryposis with a Novel *MYH3* Variant

**DOI:** 10.3390/genes17070762

**Published:** 2026-06-30

**Authors:** Annalidia Donato, Davide Vecchio, Caterina Marinaro, Rossella Brando, Alessia Bauleo, Elena Falcone, Daniela Concolino

**Affiliations:** 1 Pediatric Unit, Department of Science of Health, Magna Graecia University of Catanzaro, 88100 Catanzaro, Italy; 2 Rare Diseases and Medical Genetics Unit and Chromosomal Disorders and Dysmorphology Research Unit, Translational Paediatrics and Clinical Genetics Research Area, Bambino Gesù Children’s Hospital, IRCSS, 00165 Rome, Italy; 3 BIOGENET, Medical and Forensic Genetics Laboratory, 87100 Cosenza, Italy

**Keywords:** distal arthrogryposis, *MYH3*, compound heterozygosity, congenital contractures, genotype–phenotype correlation

## Abstract

Background: Arthrogryposis consists of a heterogeneous group of congenital disorders characterized by multiple joint contractures. Distal arthrogryposes (DAs) are often caused by pathogenic variants in fast-twitch muscle protein genes, including *MYH3*, nosologically linked to DA2A and DA2B. Methods: We evaluated two siblings with features suggestive of arthrogryposis through detailed clinical examination, radiographic imaging, audiological assessment, and targeted next-generation sequencing (NGS) for skeletal dysplasias and an arthrogryposis panel, including *MYH3*. Variants were confirmed by Sanger sequencing, and segregation analysis was performed in available relatives. Results: Both patients harbored three heterozygous *MYH3* variants: two maternally inherited in *cis* (c.749G>A and c.787G>T) and one paternally inherited in *trans* (c.4130_4138del), currently classified as variants of uncertain significance (VUS) and likely pathogenic, respectively. The female patient presented with a short neck, hand contractures, cubitus valgus, a widened internipple distance, and severe thoracolumbar scoliosis. The male sibling showed craniofacial dysmorphisms, more extensive musculoskeletal anomalies, and moderate-to-severe sensorineural hearing loss. The segregation analysis suggests an *MYH3* autosomal recessive inheritance pattern. Conclusions: This report broadens the phenotypic spectrum of *MYH3*-related disorders, suggesting potential involvement in atypical DA phenotypes resembling DA4 (scoliosis) and DA6 (hearing loss), for which causative genes remain unknown. Comprehensive molecular testing and segregation analysis are essential to clarify genotype–phenotype correlations in congenital contracture syndromes.

## 1. Introduction

Arthrogryposis, also known as Arthrogryposis Multiplex Congenita (AMC), encompasses a diverse group of conditions characterized by multiple congenital joint contractures and limited joint mobility present at birth. These conditions are typically classified into isolated and multiple contractures, with the latter affecting two or more body regions.

The term “arthrogryposis” is a clinical descriptor rather than a specific diagnosis and is observed in over 300 distinct syndromes [1]. One of the most recognizable forms is amyoplasia, which presents characteristic limb positioning, including internally rotated shoulders, extended elbows, flexed wrists, and equinovarus feet. Despite significant musculoskeletal involvement, most individuals have normal intelligence [2].

Distal arthrogryposes (DAs) are a subset of autosomal dominant syndromes primarily affecting the distal limbs. Unlike amyoplasia, DAs typically spare the proximal joints and lack underlying neurological or primary muscle disease [3]. They are classified based on phenotypic features, ranging from DA1, characterized by camptodactyly and clubfoot, to more complex forms such as DA2A (Freeman–Sheldon syndrome) and DA2B (Sheldon–Hall syndrome), which also present with craniofacial anomalies [4]. Additional variants include DA3 through DA10, each with unique features, such as scoliosis (DA4), hearing loss (DA6), and ophthalmoplegia (DA5) [3,4,5].

Molecular studies have revealed that pathogenic variants in genes encoding fast-twitch muscle fiber proteins, including *TNNI2*, *TNNT3*, *TPM2*, *MYH3*, and *MYH8*, are implicated in DA pathogenesis [6]. *MYH3*, encoding the embryonic myosin heavy chain, plays a central role. *MYH3* is expressed during early development and remains active postnatally in skeletal and cardiac muscle tissues [7]. Monoallelic pathogenic variants in *MYH3* are known to cause both DA2A and DA2B, with overlapping clinical presentations involving joint contractures [8]. Moreover, *MYH3* variants have been linked to contractures, pterygia, and spondylocarpotarsal fusion syndrome 1A (CPSFS1A), a condition marked by vertebral and carpal/tarsal fusions, and to contractures, pterygia, and spondylocarpotarsal fusion syndrome 1B (CPSFS1B), with both autosomal dominance and autosomal recessive inheritance [8]. These variants may disrupt TGF-β signaling, which is essential for proper muscle development and maintenance, further illustrating the complex molecular basis of these disorders [8].

Given the heterogeneity of AMC, accurate diagnosis requires a multidisciplinary approach combining clinical, radiological, and genetic assessments. Treatment strategies must be individualized, often involving coordinated orthopedic, rehabilitative, and surgical care to optimize patient outcomes.

Here, we report on a pair of siblings carrying three compound heterozygous *MYH3* variants (c.[749G>A;787G>T];[4130_4138del]) and showing the different expression of distal contractures. Based on their genotype–phenotype correlation, this report’s major aim is widening the knowledge of the phenotypic and molecular heterogeneity of *MYH3*-related disorders.

## 2. Case Reports

Two siblings, a six-year-old girl and a thirteen-month-old boy, born to non-consanguineous healthy parents of Moroccan origin, were referred to our clinic due to congenital musculoskeletal abnormalities. Family history was non-contributory, with no similar features or developmental disorders among relatives. Fetal movements were reported to be normal. Both were born at term after an uneventful pregnancy and normal delivery, with birth weight, length, and head circumference within the normal centiles for gestational age. At clinical examination, distal arthrogryposes, as well as mild deviation in the spinal column in the female patient and bilateral camptodactyly in the male patient, were reported without further description. No other documentation was available prior to our evaluation.

On physical examination, both presented with a short neck and congenital contractures affecting the hands.

**Figure 1:**
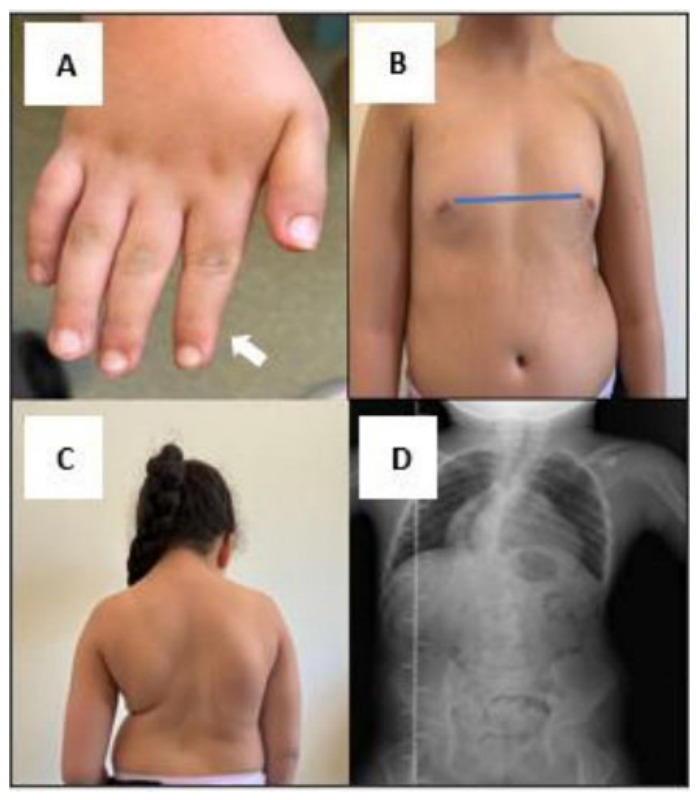
Female patient at 6 years: (**A**) fifth-finger camptodactylty and distal phalange contracture, (**B**) widened internipple distance, and (**C**,**D**) right-convex thoracolumbar scoliosis.

The older sibling had a length at the fifth percentile and a weight between the 25th and 50th percentiles, and additional findings included bilateral single transverse palmar creases, fifth-finger camptodactyly and contracture of the distal phalanges, a triggering left thumb, cubitus valgus, and a widened internipple distance. A severe right-convex thoracolumbar scoliosis was present without skeletal malformations or vertebral fusions on radiographic study (Figure 1).

**Figure 2:**
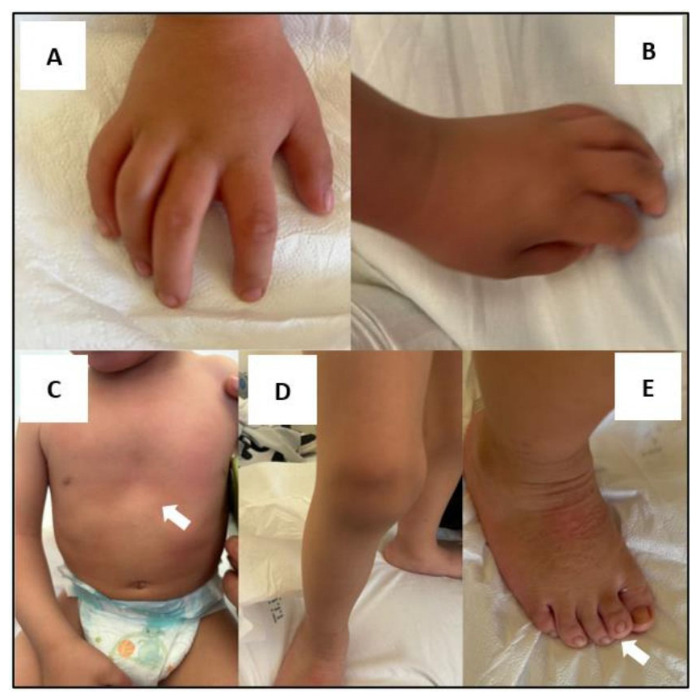
Male patient at 13 months: (**A**,**B**) bilateral polycamptodactyly, (**C**) pectus excavatum, (**D**) genu varum and bilateral knee flexion contractures, and (**E**) overlapping second toe.

The male infant had a length and weight at the 50th percentile and exhibited low-set ears, bilateral ulnar deviation of the hands and first thumbs, bilateral polycamptodactyly, pectus excavatum, genu varum and bilateral knee flexion contractures, and overlapping of the second toe onto the first (Figure 2). Audiological examination and auditory brainstem response (ABR) evaluation revealed moderate-to-severe bilateral sensorineural hearing loss.

Neuropsychiatric evaluation showed IQ in the normal range; neurological examination and electromyography, aCGH, and molecular analysis for skeletal dysplasia were normal in both patients. Given the clinical suspicion of AMC, molecular analysis was performed using a next-generation sequencing (NGS) panel for skeletal dysplasias and arthrogryposis.

## 3. Molecular Analysis

Genomic DNA was extracted from peripheral blood, followed by next-generation sequencing (NGS). Library preparation was performed using Clinical Exome Solution (Clinical Exome_Exp_v2, CNVs available) by Twist (South San Francisco, CA, USA), a target enrichment workflow that targets 6700 known disease-associated genes, according to the manufacturer’s instructions. Library quality and quantity were assessed using D1000 and High-Sensitivity D1000 Reagent Kits on a TapeStation 4150 system (Agilent Technologies, Santa Clara, CA, USA). Normalized libraries were sequenced on an Illumina NextSeq platform using a NextSeq 500/550 Mid Output Kit v2.5 (300 cycles) (Illumina, San Diego, CA, USA). Base calling and quality scoring were performed using the manufacturer’s software Real-Time Analysis v.1.18.54, followed by the generation of FASTQ sequence files. Reads were mapped to the GRCh38 reference genome (mean target coverage: 125.9821, target regions 100X: 70.34% for male sibling; mean target coverage: 141.08, target regions 100X: 81.41% for female sibling). A virtual panel for skeletal dysplasias and arthrogryposis (Supplementary Table S1) was applied for the secondary bioinformatic analysis. Variant annotation, filtering, and interpretation were performed using Expert Variant Interpreter—eVai (enGenome, Pavia, Italy) according to the American College of Medical Genetics and Genomics (ACMG) guidelines and ACGS best practice guidelines for variant classification in rare diseases [9,10]. Variants identified in probands and in their relatives were confirmed by Sanger sequencing using the primers in Supplementary Table S2. Sequencing reactions were performed using the BigDye™ Terminator v.3.1 Cycle Sequencing Kit (Applied Biosystems, Life Technologies, Waltham, MA, USA), followed by capillary electrophoresis on a SeqStudio Genetic Analyzer (Applied Biosystems). Sequence analysis was carried out with Sequencher v5.4.6 (Gene Codes Corporation, Ann Arbor, MI, USA).

Genetic testing was performed in accordance with the Declaration of Helsinki, after obtaining written informed consent from the patients’ parents, who also authorized data publication.

## 4. Results

**Figure 3:**
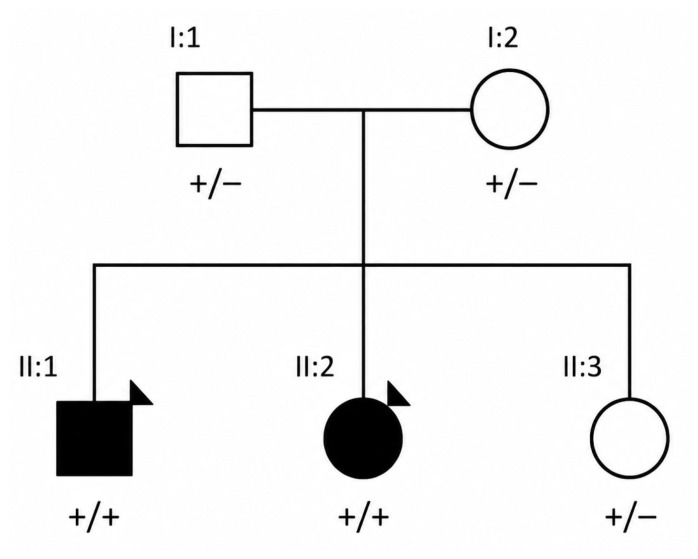
Family pedigrees. Black squares and circles represent affected individuals with distal contractures; +/+ and +/− symbols, respectively, represent compound heterozygosity (II:1 and II:2 c.[749G>A;787G>T];[4130_4138del]) and heterozygosity (I:1 c.[4130_4138del];[=], I:2 and II:3 c.[749G>A;787G>T];[=]). The arrowhead in the pedigree indicates the probands.

Genetic testing identified three heterozygous variants in the *MYH3* gene in both patients: two maternal VUS variants in *cis* (c.749G>A and c.787G>T) and a paternal, likely pathogenic variant in *trans* (c.4130_4138del) (Figure 3).

**Figure 4:**
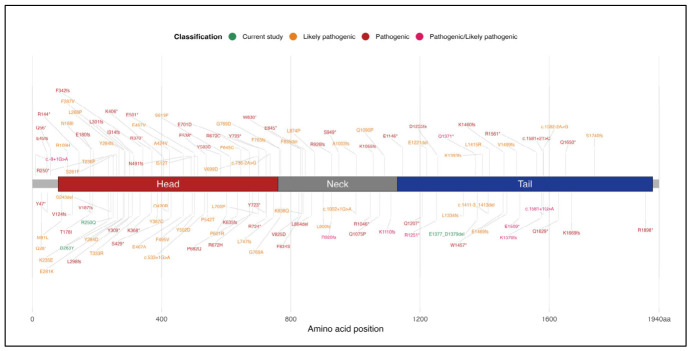
Variant distribution across MYH3 protein domains: Likely pathogenic (orange), pathogenic (brownish), and pathogenic/likely pathogenic (purple) variants identified in the literature (ClinVar database) and the three variants in the current study, D263Y, R250Q, and E1377_D1379del (green). *: stop codon.

The c.749G>A, p.(Arg250Gln) variant is a missense variant occurring in a well-established mutational hotspot of the gene, with a low population frequency in gnomAD; in silico prediction tools indicate that this variant is likely to have a deleterious effect on protein structure/function (PP2, PM2, PP3, PP1). The c.787G>T, p.(Asp263Tyr) variant is a missense alteration involving a mutational hotspot, also with a very low population frequency and predicted to have a damaging effect by in silico analysis (PP2, PM2, PP3, PP1). The c.4130_4138del p.(Glu1377_Asp1379del) variant is a novel variant, an in-frame deletion causing the loss of three amino acids in a non-repetitive region, located in an evolutionarily conserved domain, absent from public databases (PM2, PM4, PP1_Mod) (Figure 4). All three variants co-segregated with disease. According to the ACMG and ACGS criteria, the c.749G>A and c.787G>T variants can be classified as VUS, while the c.4130_4138del variant can be classified as likely pathogenic (Supplementary Table S3; Supplementary Figure S1).

No other candidate variants in arthrogryposis genes were detected.

**Figure 5:**
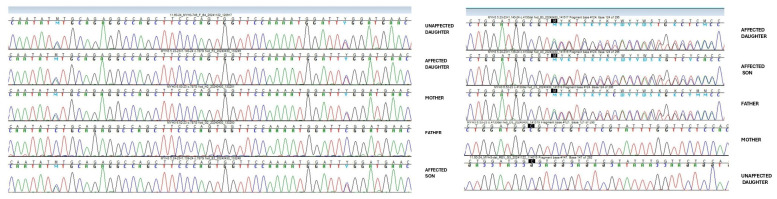
Sanger sequencing analysis of the three variants in the family. On the left, the two missense variants in *cis* (c.749G>A and c.787G>T); on the right, the in-frame deletion (c.4130_4138del).

Subsequent genetic testing of an asymptomatic sister revealed the same two maternal variants, confirming her carrier status (Figure 5).

## 5. Discussion

The present report highlights the clinical and molecular heterogeneity of *MYH3*-related arthrogryposis. The phenotypic overlap observed in both siblings, including congenital hand contractures and axial skeletal anomalies, aligns with the spectrum described for DAs. However, despite carrying the same compound heterozygous variants in *MYH3*, the two siblings exhibited distinct clinical manifestations, showing interindividual variability. The female patient presented with pronounced scoliosis and minor craniofacial involvement, while the male sibling showed more extensive musculoskeletal abnormalities and moderate-to-severe hearing loss.

In Table 1, we compare the two affected siblings with the defining features of DA2A, DA2B, DA4, DA6, and recessive *MYH3*-related CPSFS phenotypes.

However, it cannot be excluded that this different expressivity may be related to other factors, such as sex-related modifiers, additional genetic modifiers, and environmental factors.

The identification of the above-mentioned three *MYH3* variants in compound heterozygosity raises important questions regarding their individual and combined pathogenic potential. Given their segregation with disease in the two affected probands, alongside the heterozygous state retrieved in the asymptomatic parents and sister, a probable *MYH3* autosomal recessive inheritance can be hypothesized, as recently reported in the literature [11,12]. The absence of clinical manifestations in heterozygous carriers makes a fully penetrant dominant mechanism less likely, although functional studies are required to establish the precise molecular effects of the identified variants in order to elucidate whether the two variants in *cis* act synergistically and how the variant in *trans* may contribute to the overall phenotype. In this context, some authors have proposed the term *MYH3*-associated skeletal fusion syndrome (MASF) to capture this expanded clinical variability. Furthermore, these studies indicate that, given the considerable phenotypic variability observed both between and within families, drawing a clear and consistent boundary between dominant and recessive presentations may be challenging and not always straightforward from a clinical or biological standpoint [8].

From a diagnostic perspective, this report underscores the limitations of phenotype-driven classification in arthrogryposis syndromes. Although *MYH3* is a well-recognized cause of several distal arthrogryposes syndromes [8], the clinical manifestations observed in our patients differ from the classical presentations most commonly associated with *MYH3*-related disorders. In particular, the severe scoliosis observed in the female sibling and the sensorineural hearing loss identified in the male sibling resemble features that have been reported in association with DA4 and DA6, respectively. Nevertheless, these observations are based on a single family and further descriptions are necessary.

The overlapping and atypical features observed in the present cases highlight the need for comprehensive genomic approaches, particularly in patients who do not fully meet established diagnostic criteria. Early molecular diagnosis may facilitate appropriate surveillance, genetic counseling, and the anticipatory management of associated complications, including progressive spinal deformities and sensory deficits.

Moreover, this report emphasizes the importance of recognizing the wide phenotypic spectrum associated with *MYH3* variants. Notably, the observed manifestations do not fully correspond to any known subtype of distal arthrogryposis, supporting the concept that *MYH3*-associated conditions may represent a continuum rather than distinct clinical entities.

## 6. Conclusions

We report a family with arthrogryposis and variable clinical manifestations in two affected siblings carrying compound heterozygous *MYH3* variants, including two variants in *cis* (c.749G>A and c.787G>T) and one variant in *trans* (c.4130_4138del). The segregation pattern and clinical findings provide suggestive evidence of a possible genotype–phenotype association and further support the expanding phenotypic spectrum of *MYH3*-related disorders, which may be influenced also by both inheritance patterns and gene dosage effects. However, the interpretation of the identified variants remains limited by the absence of functional validation, and a causal relationship with the observed phenotype cannot be established with certainty. Additional families, detailed segregation analyses, and functional studies are needed to clarify the pathogenic role of these variants and their contribution to phenotypic variability.

## Figures and Tables

**Table 1:** Comparison of the clinical features observed in the two affected siblings with the principal distal arthrogryposis subtypes and recessive *MYH3*-related congenital pterygium, scoliosis, and vertebral fusion syndrome (CPSFS). Abbreviations: CPSFS, congenital pterygium, scoliosis, and vertebral fusion syndrome; AD, autosomal dominant; AR, autosomal recessive; O.R., occasionally reported.

Clinical Feature	Sibling 1 (Female)	Sibling 2 (Male)	DA2A	DA2B	DA3	DA4	DA5	DA6	DA7	DA8	DA9	DA10	Recessive *MYH3*- Related CPSFS
**Inheritance**	Hypothesized AR	Hypothesized AR	AD	AD	AD	AD	AD	AD	AD	AD	AD	AD	AR
**Distal limb contractures**	+	+	+	+	+	+	+	+	+	+	+	+	+
**Proximal joint involvement**	-	Knee flexion contractures	Usually absent	Usually absent	Usually absent	Usually absent	Usually absent	Usually absent	Variable	Variable	Variable	Variable	May be present
**Camptodactyly**	+	+	Typical	Typical	Typical	Common	Common	Common	Common	Common	Common	Common	Frequent
**Clubfoot**	-	-	Frequent	Frequent	Frequent	Frequent	Frequent	Frequent	Variable	Variable	Variable	Variable	Variable
**Craniofacial abnormalities**	Not identified	Low-set ears only	Distinctive facies	Mild involvement	Cleft palate may occur	Usually absent	Distinctive facies	Usually absent	Usually absent	Usually absent	Usually absent	Usually absent	Mild or absent
**Cleft palate**	-	-	O.R.	O.R.	Typical feature	Not typical	Not typical	Not typical	Not typical	Not typical	Not typical	Not typical	Rare
**Short neck**	+	+	Not typical	Not typical	Not typical	Variable	Variable	Variable	Variable	Variable	Variable	Variable	Reported
**Scoliosis**	Severe Thoracolumbar scoliosis	Not observed	Uncommon	O.R.	O.R.	Defining feature	O.R.	O.R.	O.R.	Common	Variable	Variable	Frequent
**Sensorineural hearing loss**	-	Moderate-to-severe	Not typical	Not typical	Not typical	Not typical	Not typical	Defining feature	Not typical	Not typical	Not typical	Not typical	Reported in some patients
**Ophthalmoplegia**	-	-	-	-	-	-	Defining feature	-	-	-	-	-	O.R.
**Ptosis**	-	-	-	-	-	-	Common	-	-	-	-	-	Variable
**Pectus abnormalities**	-	Pectus excavatum	O.R.	O.R.	Variable	Variable	Variable	Variable	Variable	Variable	Variable	Variable	Reported
**Pterygia**	-	-	-	-	-	-	-	-	-	Not typical	Typical	Not typical	May be present
**Vertebral fusion**	-	-	-	-	-	-	-	-	-	Typical	Variable	Variable	Typical
**Carpal/tarsal fusion**	Not detected	Not detected	-	-	-	-	-	-	-	Typical	Variable	Variable	Typical
**Neurological abnormalities**	-	-	-	-	-	-	-	-	-	-	-	-	Typically absent
**Overall phenotypic concordance with present family**	-	-	Limited	Limited	Partial	Partial overlap due to scoliosis	Limited	Partial overlap due to hearing loss	Limited	Partial	Partial	Partial	Greatest overall concordance

## Data Availability

The clinical and molecular data supporting the findings are included within the article. Additional anonymized information may be made available by the corresponding authors upon reasonable request, subject to ethical and privacy restrictions.

## Supplementary Materials

The following supporting information can be downloaded at https://www.mdpi.com/article/10.3390/genes17070762/s1. Table S1: Gene panels; Table S2: Primer Pairs, Table S3: Variants description table. Figure S1: Screenshots of the *.bam files.

## Abbreviations

The following abbreviations are used in this manuscript:

DAs	Distal arthrogryposes
aCGH	Chromosomal microarray analysis
ABR	Auditory brainstem response
VUS	Variants of uncertain significance
