# Supplementary material for: Clinical and Molecular Heterogeneity of MYH3-Related Arthrogryposis with a Novel MYH3 Variant
Source: Genes (Basel). 2026 Jun 30;17(7):762. doi: 10.3390/genes17070762 (PMC13408948; doi:10.3390/genes17070762)
Supplement: Supplementary file 1 [file genes-17-00762-s001.zip › genes-4356727-supplementary.pdf]

Supplementary Table S1: Gene panels

**Gene Panel**	**Genes**
Skeletal Dysplasia Panel	*ABCC9; ACAN; ACP5; ACTB; ACTG1; AKT1; ALDH3A2; ANKH; ANOS1; ARSB; B3GALT6; B4GALT7; BMPR1B; CANT1; CDH3; CDKN1C; CHD7; CHST3; COL10A1; COL11A1; COL11A2; COL2A1; COL3A1; COL9A1; COL9A2; COL9A3; COMP; CTSA; CTSK; DDR2; DYM; DYNC2H1; EIF2AK3; FGF8; FGFR1; FGFR2; FGFR3; FIG4; FLNA; FLNB; FUCA1; GDF5; GNAS; HBB; HDAC6; HESX1; HEXB; HMGA2; HSPG2; IDUA; IFT140; IFT172; IFT80; KCNJ8; KIF22; KIF7; KISS1R; LBR; LEMD3; LIFR; LMNA; LMX1B; MAN2B1; MATN3; MMP13; MMP9; NFIX; NKX3-2; NLRP3; NSMF; PAPSS2; PEX1; PEX10; PEX11B; PEX12; PEX13; PEX14; PEX16; PEX19; PEX2; PEX26; PEX3; PEX5; PEX6; PEX7; PHYH; PIK3CA; POLR1C; POLR1D; PROK2; PROKR2; PTEN; PTH1R; RUNX2; SDHB; SDHC; SDHD; SEMA3A; SF3B4; SLC26A2; SLC39A13; SMARCAL1; SOST; SOX10; SPRY4; TACR3; TBXAS1; TCOF1; TGFB1; TMEM165; TREM2; TRIP11; TRPV4; TTC21B; TYROBP; WDR11; WDR19; WDR35; XYLT1; ZMPSTE24*
Congenital Arthrogryposis Panel	*ACTA1; ADCY6; ALG3; ASCC1; CHST14; CNTN1; DNM2; DOK7; ERBB3; FBN2; FKBP10; FKBP14; GLE1; MYH3; MYH8; MUSK; MYBPC1; PIEZO2; PIP5K1C; PLOD2; RAPSN; SCARF2; SYNE1; TNNI2; TNNT3; TOR1A; TPM2; UBA1; VIPAS39; VPS33B*

Supplementary Table S2: Primer Pairs

MYH3<br><br>NM_002470.4	Primer forward	Primer reverse
c.749G>A; p.(Arg250Gln)<br>c.787G>T; p.(Asp263Tyr)	CCTAAAAGACCAGCCCCACT	GTGGCACGTGATTGTAGTCC
c.4130_4138del;<br>p.(Glu1377_Asp1379del)	CCCAGCACCGCATTTAAAGA	AACTTCTGCAGGCCAAGAAC

Supplementary Table S3: Variants description table

Gene<br>Transcript	Exon	c.HGVS<br><br>p.HGVS	Protein Domain	State<br><br>*Type of variant*	Reference Assembly: GRCh38 position	Classification	Criteria	Variant Fraction
MYH3<br>*NM_002470.4*	9	c.749G>A<br><br>p.(Arg250Gln)	Head Domain	Heterozygous<br><br>*Missense*	Chr17:<br>g.10647413	VUS<br>(Classe III)	PM2 gnomAD: 0,006%<br><br>ClinVar contains one entry for this variant (Variation ID: 1428406) classified as VUS.<br><br>PP3: CADD (score 33), BayesDel (0.17) suggest that this variant may disrupt the consensus splice site.<br><br>PP2: missense variant in a gene that has a low rate of benign missense variation and in which missense variants are a common mechanism of disease.<br><br>PP1: Co-segregation with disease in multiple affected family members in a gene definitively known to cause the disease.	85 (45/40)<br>52.94%
MYH3<br>*NM_002470.4*	9	c.787G>T<br><br>p.(Asp263Tyr)	Head Domain	Heterozygous<br><br>*Missense*	Chr17:<br>g.10647375	VUS<br>(Classe III)	PM2 gnomAD: 0,003%<br><br>ClinVar contains one entry for this variant (Variation ID: 1439380) classified as VUS.<br><br>PP3: CADD (score 33), BayesDel (0.36) suggest that this variant may disrupt the consensus splice site.<br><br>PP2: missense variant in a gene that has a low rate of benign missense variation and in which missense variants are a common mechanism of disease.<br><br>PP1: Co-segregation with disease in multiple affected family members in a gene definitively known to cause the disease.	107 (56/51)<br>52.33%
MYH3<br>*NM_002470.4*	30	c.4130_4138del<br><br>p.(Glu1377_Asp1379del)	Tail Domain	Heterozygous<br><br>In-frame deletion	Chr17:<br>g.10635401	Likely Pathogenic<br>(Classe IV)	PM2 gnomAD: absent<br><br>Absent in Clinvar and in literature.<br><br>PM4: disruptive in-frame deletion of 3 aa within the exon 30. In a non-repeat region.<br><br>PP1_Mod: Co-segregation with disease in multiple affected family members in a gene definitively known to cause the disease and located in an evolutionarily conserved region.	189 (84/105)<br>44.44%

Supplementary Figure S1: Screenshots of the *.bam files of *MYH3* gene, showing the two missense variants c.749G>A and c.787G>T and the in-frame deletion c.4130_4138del in the two siblings.

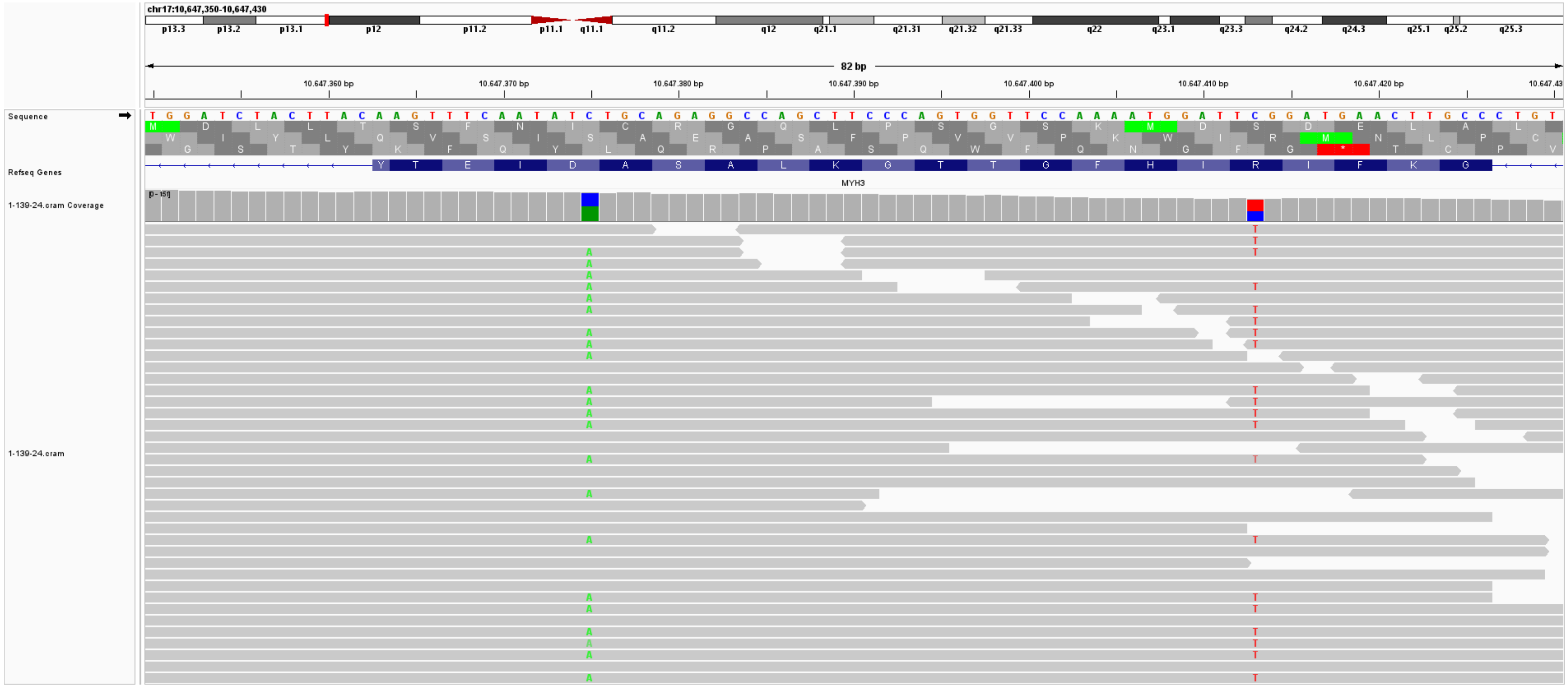

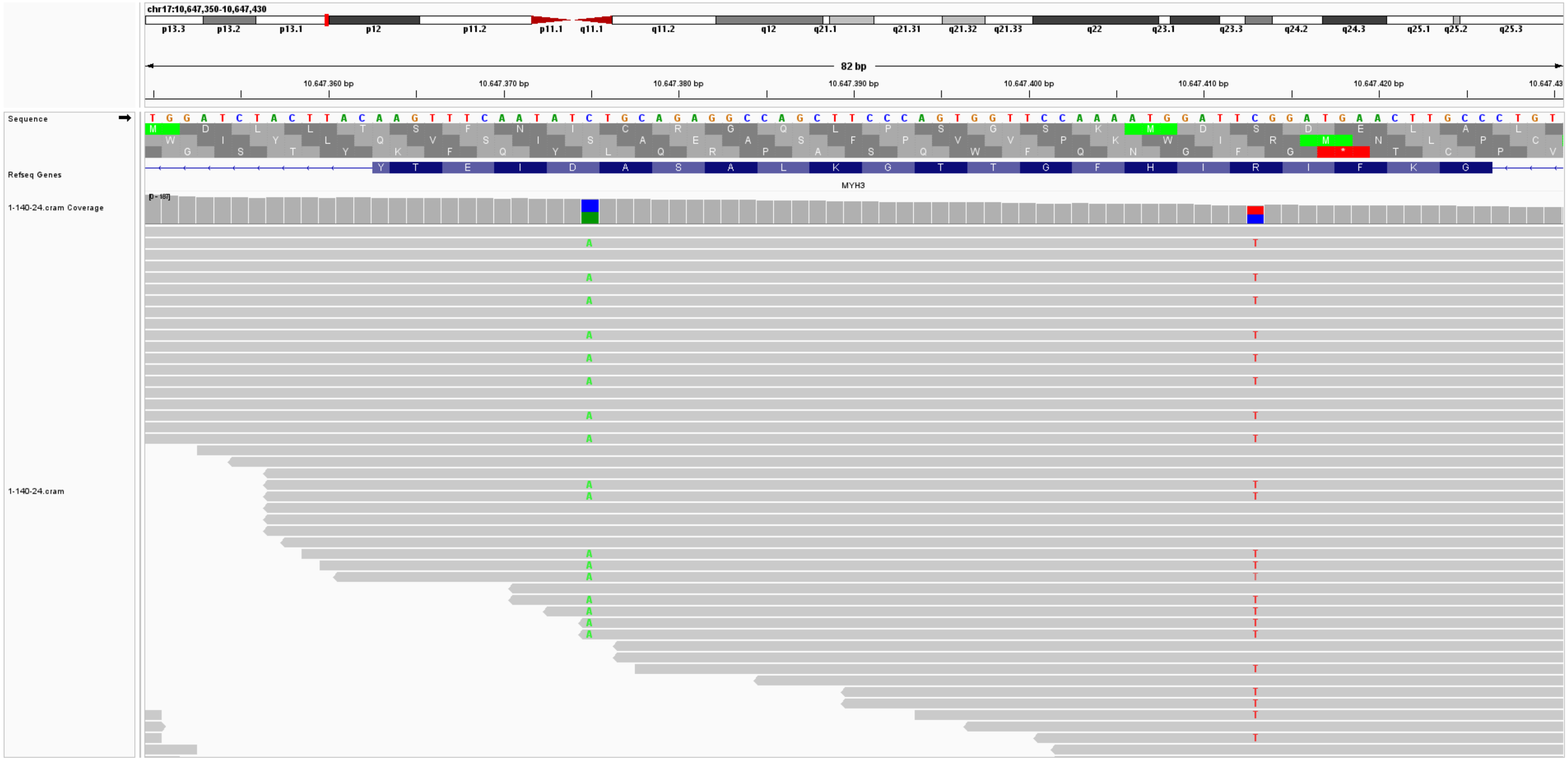
chr17:10,647,350-10,647,430
82 bp
Sequence
Refseq Genes
MYH3
1-140-24.cram Coverage
1-140-24.cram

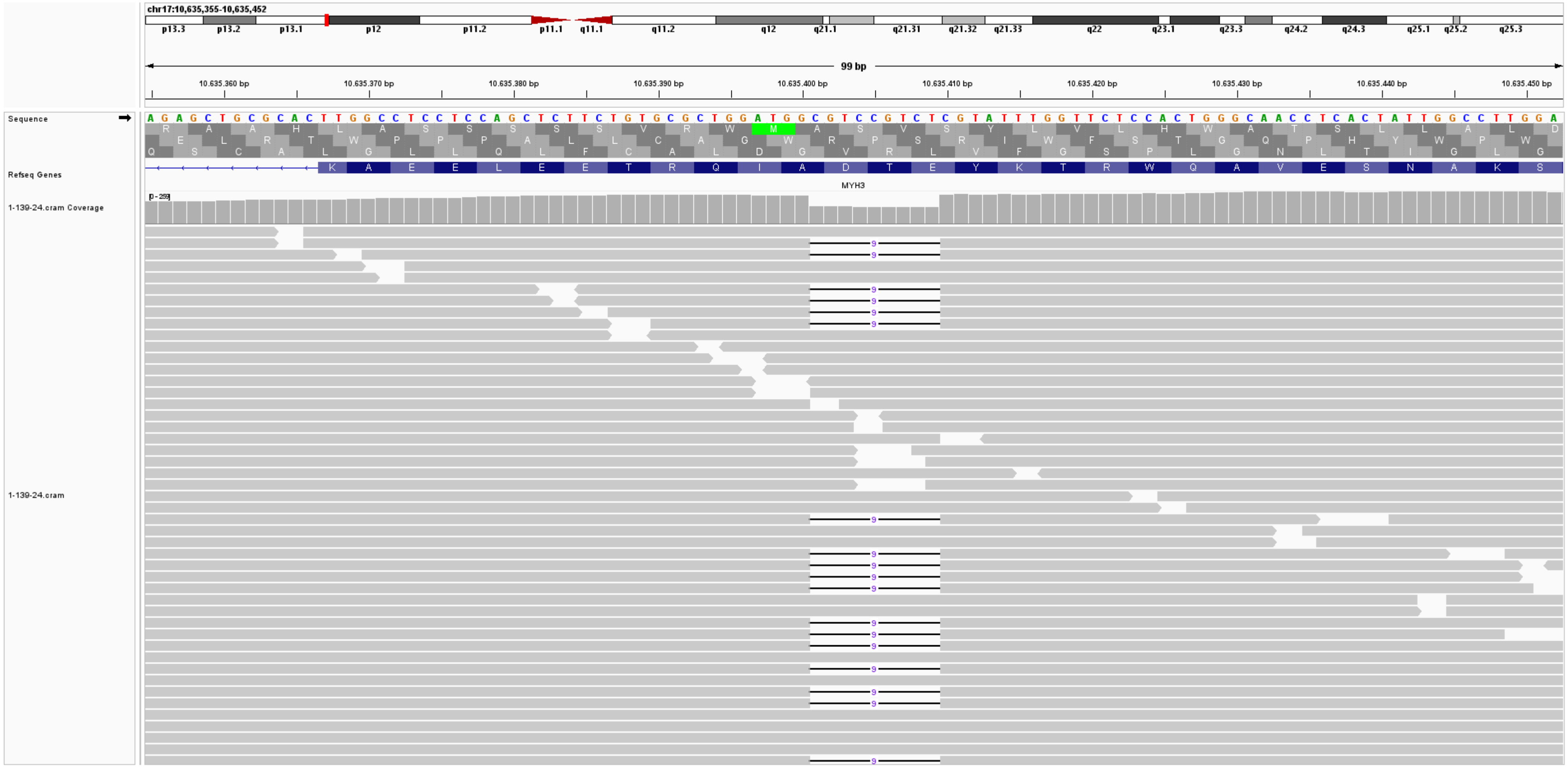
chr17:10,635,355-10,635,452
99 bp
10.635.360 bp
10.635.370 bp
10.635.380 bp
10.635.390 bp
10.635.400 bp
10.635.410 bp
10.635.420 bp
10.635.430 bp
10.635.440 bp
10.635.450 bp
Sequence
Refseq Genes
MYH3
1-139-24.cram Coverage
1-139-24.cram

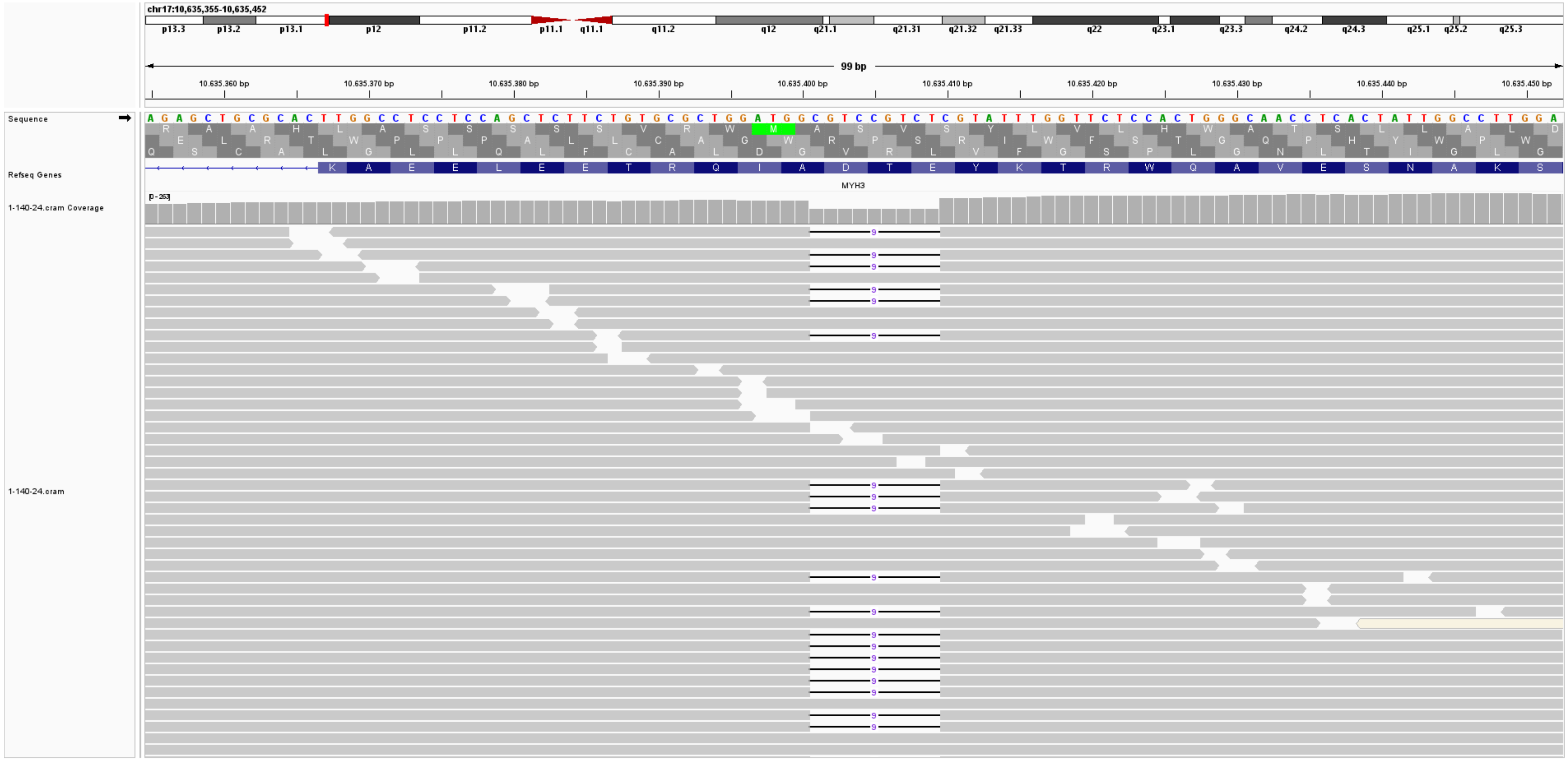

chr17:10,635,355-10,635,452
p13.3
p13.2
p13.1
p12
p11.2
p11.1
q11.1
q11.2
q12
q21.1
q21.31
q21.32
q21.33
q22
q23.1
q23.3
q24.2
q24.3
q25.1
q25.2
q25.3
99 bp
10.635.360 bp
10.635.370 bp
10.635.380 bp
10.635.390 bp
10.635.400 bp
10.635.410 bp
10.635.420 bp
10.635.430 bp
10.635.440 bp
10.635.450 bp
Sequence
Refseq Genes
MYH3
1-140-24.cram Coverage
[0-263]
1-140-24.cram
